# Survival in Papillary Thyroid Microcarcinoma: A Comparative Analysis Between the 7th and 8th Versions of the AJCC/UICC Staging System Based on the SEER Database

**DOI:** 10.3389/fendo.2019.00010

**Published:** 2019-01-24

**Authors:** Fan Yang, Qi Zhong, Zhigang Huang, Meng Lian, Jugao Fang

**Affiliations:** ^1^Department of Otorhinolaryngology, Head and Neck Surgery, Beijing Tongren Hospital, Capital Medical University, Beijing, China; ^2^Department of Otorhinolaryngology, Head and Neck Surgery, Beijing Anzhen Hospital, Capital Medical University, Beijing, China

**Keywords:** papillary thyroid microcarcinoma, AJCC/UICC staging system, surveillance, Epidemiology and End Results, survival, prognostic efficacy

## Abstract

**Background:** Considerable modifications have been introduced in the new edition of the American Joint Committee on Cancer/Union for International Cancer Control (AJCC/UICC) TNM staging system. Based on the National Cancer Institute's Surveillance, Epidemiology, and End Results (SEER) database, this study aimed to compare the 7th and 8th editions of the AJCC/UICC TNM staging system for patients with papillary thyroid microcarcinoma (PTMC) and follicular variant papillary thyroid microcarcinoma (FVPTMC).

**Methods:** A Data from 2004 to 2014 of 39,032 patients registered in the SEER database were included. The 7th and 8th editions of the AJCC/UICC staging system were compared in terms of TNM staging, age cutoff, and clinical staging. Patient survival was evaluated using Kaplan-Meier and multivariable Cox proportional hazards models. The American Thyroid Association (ATA) risk stratification system was integrated with the AJCC/UICC staging system for further investigation. Receiver operating characteristic (ROC) curves, Harrell's C-index, Akaike information criterion (AIC), and the Bayesian information criterion (BIC) were used to assess the models' performances.

**Results:** Revised TNM categories, age cutoff, and clinical staging in the 8th edition resulted in reclassification of the overall stage. Applying the 8th edition, 1,278 stage III and 425 stage IV patients were reclassified as stage I; 950 stage III and 459 stage IV patients were reclassified as stage II; 77 stage IV patients were reclassified as stage III; and only 88 patients remained in stage IV. All patients in stage I, according to the 7th edition, remained in this stage when using the 8th edition. Patients classified into higher stages (III and IV) in the 8th edition showed a worse prognosis than those classified into same stages in the 7th edition. The 8th edition proved to be a better model with higher prognostic efficacy survival (higher AUC and C-index, lower AIC and BIC) than the 7th edition. When integrated with the ATA risk stratification system, the 8th edition still showed better discriminative power for patients in the higher risk group.

**Conclusion:** Based on the SEER database, the 8th edition of the AJCC/UICC staging system has better prognostic efficacy than the 7th edition for patients with PTMC and FVPTMC.

## Introduction

Thyroid cancer is one the most common endocrine malignancies and its incidence is rapidly increasing (1, 2). Most patients are diagnosed with papillary thyroid carcinoma (PTC) and approximately half of cases are identified as papillary thyroid microcarcinoma (PTMC), most of whom are classified as typical PTMC or follicular variant papillary thyroid microcarcinoma (FVPTMC) (2, 3). According to the World Health Organization (WHO) classification, PTMCs are small thyroid tumors with a maximum diameter of 1 cm (4). Papillary thyroid microcarcinoma usually has favorable prognosis and is often regarded as an indolent malignancy; however, lymph node metastasis (LNM) or local and/or distant recurrence may occur in the minority of patients, even after surgery or radioactive iodine treatment, leading to worse prognosis (5, 6).

The American Joint Committee on Cancer/Union for International Cancer Control (AJCC/UICC) updated its tumor node metastasis (TNM) staging system and published the 8th edition in 2018, in which conspicuous changes were introduced, reflecting a better understanding of the clinicopathological factors combined with cancer-specific survival regarding thyroid cancer (7). The main changes include an increased age cutoff, the new definition of the T3 and N categories and the updated clinical staging definition (Supplementary Table 1). As a result, a large proportion of patients with advanced stages (stages III and IV), according to the 7th edition of the AJCC/UICC TNM staging system, are now classified into earlier stages (stages I and II) (7, 8).

To further investigate the impact of the new TNM staging system on PTMC and FVPTMC, and compare it with the previous 7th edition, we conducted a retrospective analysis using data from the National Cancer Institute's Surveillance, Epidemiology, and End Results (SEER) Program.

## Materials and Methods

### Data Source and Study Subjects

We conducted a retrospective cohort analysis using the SEER database from 2004 to 2014. Patients with confirmed histological diagnosis of papillary thyroid cancer (International Classification of Diseases for Oncology (ICD-O) code C73.9) were selected and the definition of papillary thyroid microcarcinoma relied on the tumor size code 001-010 (millimeters (mm) and 991 [described as “less than 1 centimeter (cm)”]. The histologic subtypes were selected as follows: 8050/3 (papillary carcinoma, NOS). 8260/3 (papillary adenocarcinoma, NOS), 8340/3 (papillary carcinoma, follicular variant), 8341/3 (papillary microcarcinoma), 8343/3 (papillary carcinoma, encapsulated).

Demographic data included sex, age at diagnosis, year of diagnosis and ethnicity. The cancer characteristics included tumor extension, lymph node metastasis, distant metastasis, and TNM/clinical stage in the 7th AJCC/UICC staging system. The TNM stage in the 8th AJCC/UICC staging system was defined according to the SEER data of tumor extension, lymph node metastasis, and distant metastasis (Supplementary Table 2). The clinical stage of patients was then defined according to the TNM stage in the 8th AJCC/UICC staging system. All variables were defined using the SEER specific codes. Stage T2 (in both editions) and T3a (in the 8th edition) were excluded as they do not meet the definition criteria of thyroid microcarcinoma (tumor size with <1 cm). Patients with unknown variables (code 999) whose stage could not be classified were excluded.

Although the American Thyroid Association (ATA) risk stratification system was designed to provide estimation of the risk of disease recurrence, it still includes several variables that may affect cancer-specific survival (9). Thus, we also integrated the ATA risk stratification system with the two editions of the AJCC/UICC system. We used the 2009 ATA risk stratification system, which partially differs from the most recent updated version, due to limitations in the available clinicopathological data in the SEER database (10).

### Statistical Methods

Numerical data were expressed as mean ± standard deviation, and categorical data were expressed as percentages. The chi-square and Fisher's exact tests were used to evaluate the relationship between clinical characteristics. Survival was estimated using the Kaplan-Meier method and comparisons between groups were made using a log-rank test. The effects of potential predictors of overall survival (OS) and cancer-specific survival (CSS) were assessed using Cox proportional hazards regression and reported as hazard ratios (HRs) with 95% confident intervals (CIs). Receiver operating characteristic (ROC) curves and the Harrell's C concordance index (C-index), a method used for assessing the probability of concordance between expected and observed outcomes, was used to evaluate the prognostic efficacy of CSS of the 7th and 8th AJCC/UICC staging systems using stage-determinant variables (age, T, N, and M categories) (11). In order to measure the relative quality of the two editions of the AJCC/UICC staging system, we used the Akaike information criterion (AIC) and the Bayesian information criterion (BIC). AIC and BIC served as standards to measure the quality of model fitness by providing asymptotically unbiased estimators between the true model and the fitted approximating model (12, 13). In summary, the model with higher C-index and lower AIC and BIC is considered to have better prognostic efficacy. A *p*-value lower than 0.05 was considered statistically significant. Statistical analyses were performed using SPSS version 25.0 (IBM Corp., Armonk, NY, USA) and R statistical software v3.5.1 (The R Project for Statistical Computing, Vienna, Austria) with package Survival (14). ECharts 4.0 (Baidu Corp., Beijing, China), an open-source data visualization software, was used to draw the alluvial flow diagram.

### Ethical Statement

This retrospective study used data from the SEER database, which is designed and maintained by the National Cancer Institute. The research was limited to the secondary use of previously collected information and data were anonymized before statistical analyses. The study was approved by the ethical review board of the Beijing Tongren Hospital, Capital Medical University, and complied with the ethical standards of the Declaration of Helsinki, as well as with relevant national and international guidelines.

## Results

### Demographic and Follow-Up Data

Our study involved 39,032 patients, out of whom 27,830 had PTMC and 11,202 had FVPTMC. All patients were staged according to the 7th AJCC/UICC staging system, which was compared to the recently published 8th edition. Detailed demographic data and follow-up information of patients are shown in Table 1. The 10-year OS of all patients was 90.9%, and the 10-year CSS was 99.4%. Patients with PTMC showed longer but not statistically different 10-year OS than those with FVPTMC (90.9 vs. 91.1%, *p* = 0.718), and showed same 10-year CSS with FVPTMC patients (both 99.4%, *p* = 0.796).

**Table 1:** Numerical data and follow-up information for patients with PTMC or FVPTMC.

**Variables**		**Overall**	**Histological types**		***p*-Value**
		**(*n* = 39,032)**	**PTMC (*n* = 27,830)**	**FVPTMC (*n* = 11,202)**	
Age (*n* ±*sv*)		51.05 ± 14.00	50.43 ± 41.03	52.59 ± 13.80	<0.01
Sex	Female	31,824 (81.5%)	22,587 (81.2%)	9,237 (82.5%)	<0.01
	Male	7,208 (18.5%)	5,243 (18.8%)	1,965 (17.5%)	
Race	Caucasian	32,505 (83.3%)	23,105 (83.0%)	9,400 (83.9%)	<0.01
	Black	2,578 (6.6%)	1,657 (6.0%)	921 (8.2%)	
	Other	3,949 (10.1%)	3,068 (11.0%)	881 (7.9%)	
Age subgroup	<45-years-old	12,767 (32.7%)	9,584 (34.4%)	3,183 (28.4%)	<0.01
	45 to 54-years-old	10,400 (26.2%)	7,454 (26.8%)	2,946 (26.3%)	
	≥55-years-old	15,865 (40.6%)	10,792 (38.8%)	5,073 (45.3%)	
Follow-up months (*n* ±*sv*)		63.13 ± 36.62	63.81 ± 36.34	61.45 ± 37.24	<0.01
	10-years OS	90.9%	91.1%	90.2%	0.718
	10-years CSS	99.4%	99.4%	99.4%	0.796

### Changing of Patients' TNM Stages

The definition of TNM stages were modified in the 8th edition. Revision of the T category in the 8th edition resulted in reclassification of almost half of T3 categories (1,078/2,063, 52.3%) to T1a, due to the removal of minimal extrathyroidal extension to the perithyroidal tissue as a standard for the T3 category, in relation to the 7th edition. Thus, the proportion of T3 patients decreased from 5.3 to 2.5%, while the proportion of T1a patients increased from 94.1 to 97.1% in the 7th and 8th editions, respectively.

Based on the 7th edition, the change of the T categories resulted in increased hazard ratios (HRs) of OS and CSS, either in univariate or multivariate analyses. Exceptions were observed when comparing the HRs of T1a and T3, as no significant statistical differences were found in multivariate analyses of both OS and CSS, and univariate analysis of OS (but were found in univariate analysis of CSS). As for the 8th edition, similar results were observed, as increased T categories led to increased HRs (the same exceptions were found when comparing the HRs of OS and CSS between T1a and T3) (Table 2).

**Table 2A:** Univariate and multivariate analysis for patient survival according to the 7th AJCC/UICC staging system.

**Variables**		**OS**				**CSS**			
		**Univariate analysis**		**Multivariate analysis**		**Univariate analysis**		**Multivariate analysis**	
		**Hazard ratio (95%CI)**	***p-*Value**	**Hazard ratio (95%CI)**	***p-*Value**	**Hazard ratio (95%CI)**	***p-*Value**	**Hazard ratio (95%CI)**	***p-*Value**
Age		1.084 (1.080–1.088)	<0.01	1.089 (1.083–1.096)	<0.01	1.089 (1.074–1.105)	<0.01	1.085 (1.062–1.109)	<0.01
Sex	Female	Reference		Reference		Reference		Reference	
	Male	2.280 (2.065–2.516)	<0.01	1.661 (1.500–1.839)	<0.01	3.040 (2.147–4.306)	<0.01	1.541 (1.067–2.226)	<0.05
Race	Caucasian	Reference		Reference		Reference		Reference	
	Black	1.476 (1.257–1.733)	<0.01	1.646 (1.400–1.934)	<0.01	1.092 (0.553–2.154)	0.801	1.110 (0.562–2.192)	0.763
	Other	0.658 (0.542–0.797)	<0.01	0.749 (0.617–0.909)	<0.01	1.126 (0.645–1.964)	0.676	1.132 (0.648–1.976)	0.663
Age subgroup	<45-years-old	Reference		Reference		Reference		Reference	
	≥45-years-old	6.233 (5.230–7.429)	<0.01	0.749 (0.601–0.933)	<0.05	9.000 (4.203–19.270)	<0.01	6.501 (2.621–13.971)	<0.01
T stage	T1a	Reference		Reference		Reference		Reference	
	T3	0.850 (0.677–1.067)	0.161	0.778 (0.595–1.017)	0.066	2.478 (1.418–4.333)	<0.01	1.361 (0.754–2.458)	0.307
	T4a	2.179 (1.386–3.424)	<0.01	1.806 (1.140–2.862)	<0.05	13.522 (6.286–29.087)	<0.01	4.501 (1.959–10.344)	<0.01
	T4b	4.224 (2.394–7.453)	<0.01	2.780 (1.543–5.009)	<0.01	53.128 (26.868–105.055)	<0.01	9.243 (4.074–20.971)	<0.01
N stage	N0	Reference		Reference		Reference		Reference	
	N1a	0.885 (0.711–1.101)	0.272	0.881 (0.707–1.098)	0.258	2.676 (1.519–4.716)	<0.01	2.248 (1.262–4.005)	<0.01
	N1b	1.778 (1.472–2.147)	<0.01	1.597 (1.307–1.951)	<0.01	9.157 (6.090–13.768)	<0.01	4.357 (2.605–7.287)	<0.01
	N1NOS	0.569 (0.296–1.097)	0.092	0.479 (0.248–0.926)	<0.05	3.852 (1.216–12.201)	<0.05	1.922 (0.592–6.241)	0.277
M stage	M0	Reference		Reference		Reference		Reference	
	M1	7.385 (5.116–10.660)	<0.01	6.238 (4.228–9.203)	<0.01	71.121 (43.702–115.742)	<0.01	22.800 (12.343–42.117)	<0.01
Clinical stage	I	Reference		Reference		Reference		Reference	
	II	2.315 (0.746–7.185)	0.146	10.674 (3.397–33.538)	<0.01	16.506 (2.294–118.786)	<0.01	0.104 (0.014–0.788)	<0.05
	III	1.194 (0.979–1.455)	0.079	0.935 (0.767–1.141)	0.257	2.603 (1.380–4.911)	<0.01	2.262 (0.986–5.192)	<0.05
	IVa	2.657 (2.172–3.250)	<0.01	1.618 (1.319–1.985)	<0.01	12.752 (7.974–20.393)	<0.01	7.453 (3.471–16.004)	<0.01
	IVb	6.490 (3.485–12.086)	<0.01	4.596 (2.465–8.568)	<0.01	97.291 (44.774–211.409)	<0.01	61.090 (11.287–330.655)	<0.01
	IVc	10.695 (7.257–15.762)	<0.01	4.245 (2.864–6.291	<0.01	145.602 (86.700–244.522)	<0.01	43.193 (20.117–92.740)	<0.01
Histological subtype	PTC	Reference		Reference		Reference		Reference	
	FVPTC	1.147 (1.037–1.269)	<0.01	1.037 (0.938–1.147)	0.479	0.925 (0.629–1.362)	0.694	0.929 (0.631–1.368)	0.708
Year of diagnosis		0.996 (0.977–1.017)	0.732	0.982 (0.963–1.002)	0.083	0.938 (0.877–1.003)	0.061	0.937 (0.876–1.003)	0.059

Out of 1532 patients classified as N1b according to the 7th edition, 154 (10.1%) patients were reclassified into the N1a stage according to the 8th edition, as level VII lymph node metastasis without lateral cervical lymph node metastasis is now classified as N1a. Therefore, the proportion of N1b patients decreased from 3.9 to 3.5%, while the proportion of N1a patients increased from 6.1 to 6.5%.

As shown in Table 2, according to both editions, significant differences in the HRs of OS were only observed between N0 and N1b patients, whereas no statistically significant difference was observed between N0 and N1a patients. Regarding CSS, increased N categories yielded dramatically increased HRs, both between N0 and N1a and between N0 and N1b patients.

Survival analysis revealed that older patients had worse prognosis than younger patients, either regarding OS or CSS. With an age cutoff of 45 years old in the 7th edition, older patients had a worse HR than younger patients (Table 2A). As the age cutoff changed to 55 years old in the 8th edition, up to 10,400 patients were moved to the younger group (<55 years old), nearly 15% of whom were in advanced clinical stages: 8,971 (86.3%) in stage I, 988 (9.5%) in stage III and 441 (4.2%) in stage IV. This age cutoff was still associated with OS and CSS, as significant differences in the HRs of OS and CSS were observed between the two age subgroups (<55 years old and ≥55 years old) (Table 2B).

**Table 2B:** Univariate and multivariate analysis for patients' survival according to the 8th AJCC/UICC staging system.

**Variables**		**OS**				**CSS**			
		**Univariate analysis**		**Multivariate analysis**		**Univariate analysis**		**Multivariate analysis**	
		**Hazard ratio (95%CI)**	***p-*Value**	**Hazard ratio (95%CI)**	***p-*Value**	**Hazard ratio (95%CI)**	***p-*Value**	**Hazard ratio (95%CI)**	***p-*Value**
Age		1.084 (1.080–1.088)	<0.01	1.082 (1.078–1.086)	<0.01	1.089 (1.074–1.105)	<0.01	1.090 (1.075–1.106)	<0.05
Sex	Female	Reference		Reference		Reference		Reference	
	Male	2.280 (2.065–2.516)	<0.01	1.825 (1.651–2.017)	<0.01	3.040 (2.147–4.306)	<0.01	1.602 (1.103–2.326)	<0.05
Race	Caucasian	Reference		Reference		Reference		Reference	
	Black	1.476 (1.257–1.733)	<0.01	1.631 (1.388–1.917)	<0.01	1.092 (0.553–2.154)	0.801	1.500 (0.754–2.985)	0.248
	Other	0.658 (0.542–0.797)	<0.01	0.757 (0.624–0.919)	<0.01	1.126 (0.645–1.964)	0.676	1.127 (0.644–1.972)	0.676
Age subgroup	<55-years-old	Reference		Reference		Reference		Reference	
	≥55-years-old	5.531 (4.946–6.184)	<0.01	4.895 (4.369–5.484)	<0.01	6.991 (4.492–10.883)	<0.01	7.256 (4.625–11.382)	<0.01
T stage	T1a	Reference		Reference		Reference		Reference	
	T3b	0.921 (0.680–1.246)	0.593	0.858 (0.631–1.166)	0.327	2.737 (1.335–5.613)	<0.01	1.480 (0.705–3.107)	0.300
	T4a	2.191 (1.394–3.444)	<0.01	1.897 (1.199–3.000)	<0.01	13.126 (3.110–28.196)	<0.01	4.852 (2.144–10.984)	<0.01
	T4b	4.248 (2.408–7.495)	<0.01	2.942 (1.636–5.291)	<0.01	51.572 (26.121–101.821)	<0.01	10.797 (4.810–24.240)	<0.01
N stage	N0	Reference		Reference		Reference		Reference	
	N1a	1.070 (0.882–1.298)	0.493	1.207 (0.845–1.249)	0.790	4.472 (2.859–6.996)	<0.01	3.397 (2.134–5.410)	<0.01
	N1b	1.556 (1.260–1.922)	<0.01	1.352 (1.084–1.686)	<0.01	6.702 (4.152–10.818)	<0.01	2.848 (1.578–5.139)	<0.01
	N1NOS	0.569 (0.296–1.096)	0.092	0.464 (0.240–0.897)	<0.05	3.847 (1.215–12.184)	<0.05	1.737 (0.533–5.662)	0.360
M stage	M0	Reference		Reference		Reference		Reference	
	M1	7.385 (5.116–10.660)	<0.01	4.569 (3.098–6.738)	<0.01	71.121 (43.702–115.742)	<0.01	16.985 (9.337–30.899)	<0.01
Clinical stage	I	Reference		Reference		Reference		Reference	
	II	2.675 (2.257–3.171)	<0.01	1.270 (1.068–1.511)	<0.01	10.491 (6.851–16.064)	<0.01	5.021 (3.232–7.798)	<0.01
	III	4.176 (2.468–7.068)	<0.01	1.802 (1.063–3.053)	<0.05	24.393 (8.931–66.623)	<0.01	10.809 (3.926–29.755)	<0.01
	IVa	6.851 (3.261–14.395)	<0.01	3.736 (1.776–7.860)	<0.01	119.595 (52.118–274.437)	<0.01	60.313 (26.171–138.998)	<0.01
	IVb	11.686 (7.596–17.979)	<0.01	3.603 (2.327–5.578)	<0.01	142.588 (80.535–252.453)	<0.01	45.712 (24.901–83.915)	<0.01
Histological subtype	PTC	Reference		Reference		Reference		Reference	
	FVPTC	1.147 (1.037–1.269)	<0.01	1.096 (0.991–1.213)	0.074	0.925 (0.629–1.362)	0.694	1.105 (0.747–1.635)	0.617
Year of diagnosis		0.996 (0.977–1.017)	0.732	0.988 (0.969–1.009)	0.258	0.938 (0.877–1.003)	0.061	0.927 (0.866–0.993)	0.052

### Changing of Clinical Stages

**Figure 1:**
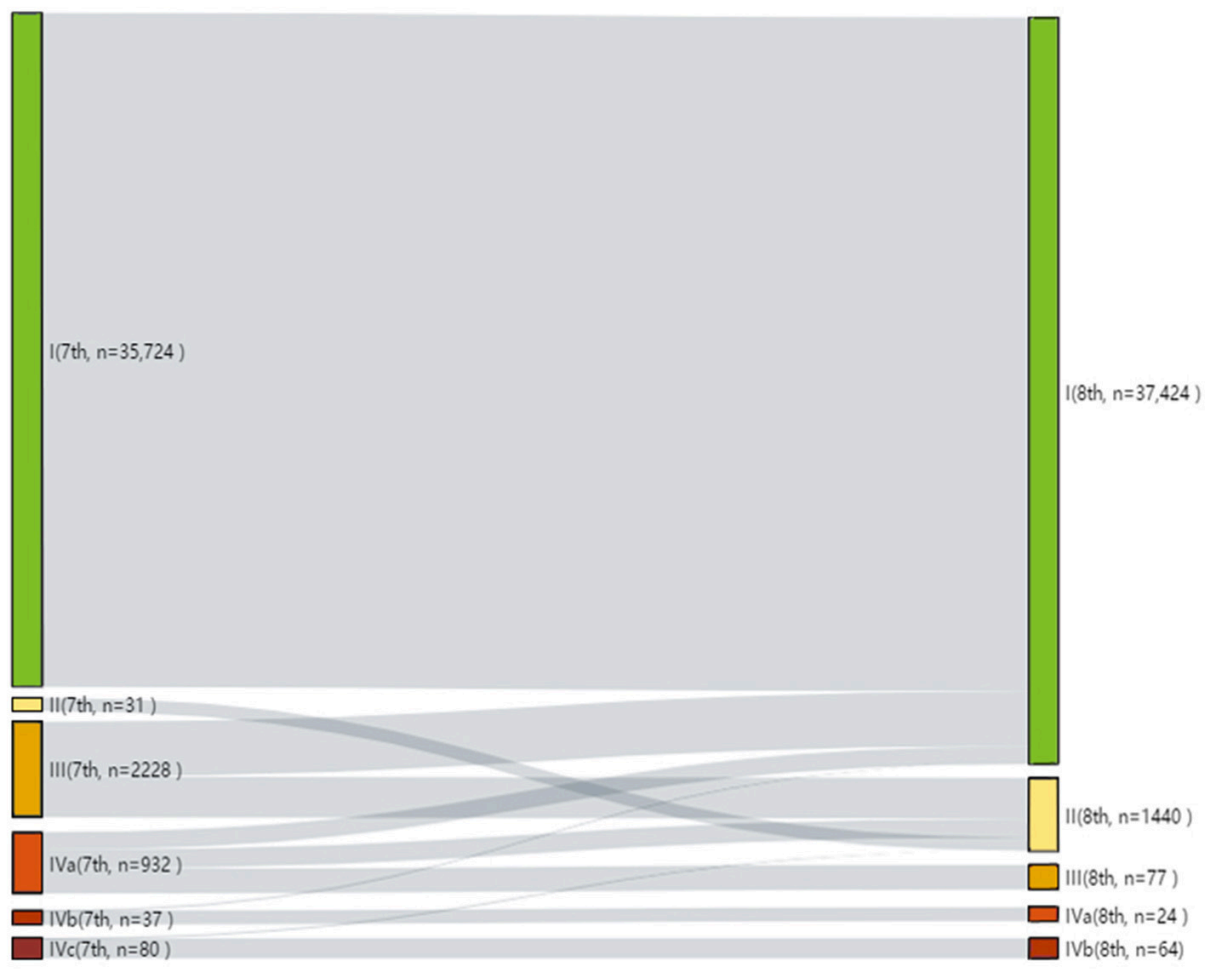
Alluvial flow diagram representing the re-staging of patient cohorts from the 7th to the 8th edition of the AJCC/UICC TNM staging system.

**Figure 2:**
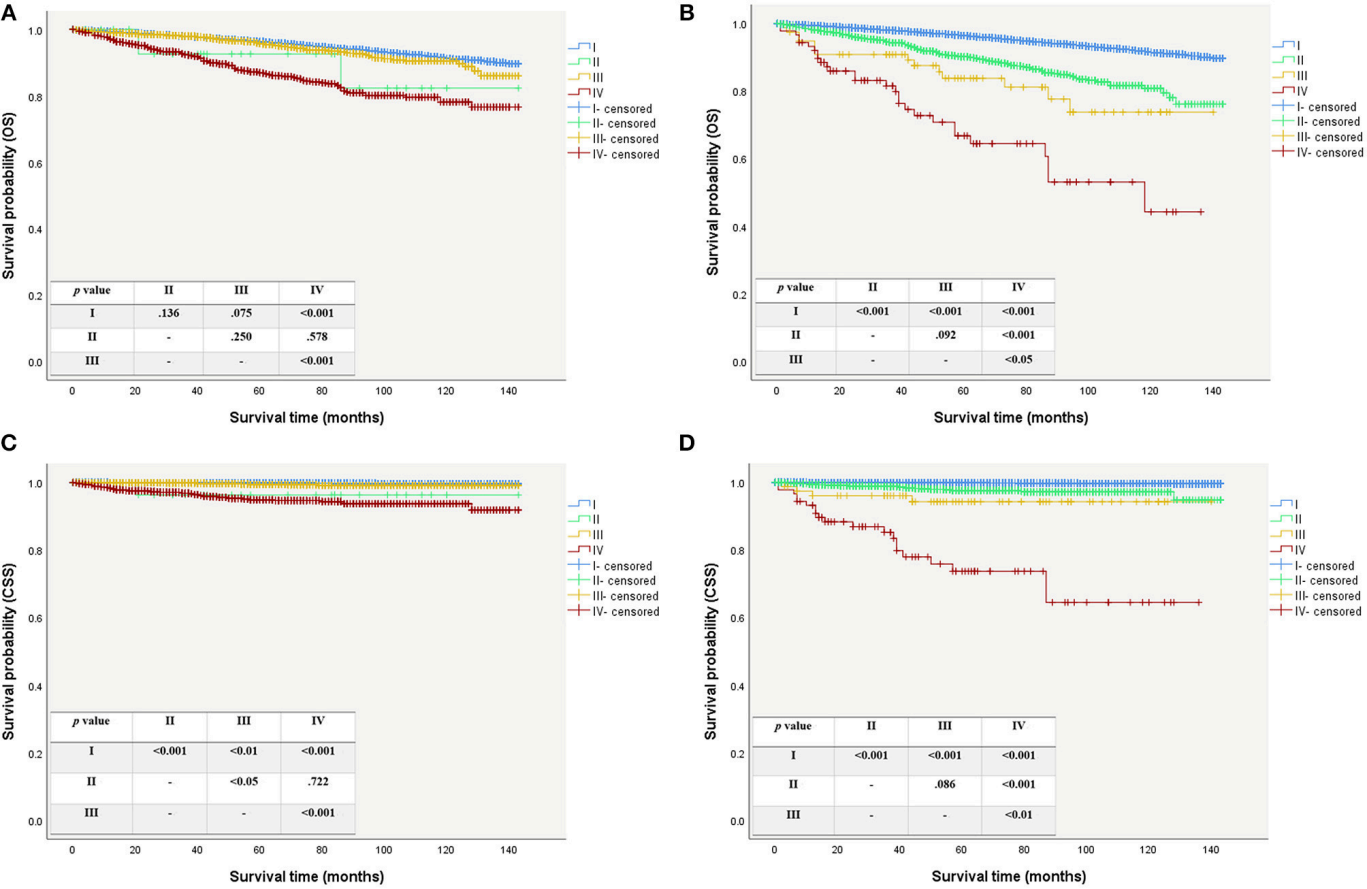
Survival curves for patient using the AJCC/UICC staging system. **(A)** Overall survival curves using the 7th edition. **(B)** Overall survival curves using the 8th edition. **(C)** Caner specific survival curves using the 7th edition. **(D)** Caner specific survival curves using the 8th edition.

Modification of the T and N categories and age cutoff led to conspicuous changes in the patients' clinical stage (Figure 1). The Kaplan-Meier survival curves showed a better separation of the stage curves in the 8th edition, when compared to the 7th edition (Figure 2). A better 10-year OS of patients with stage I in the 8th edition was observed, when compared to the same stage in the 7th edition, although patients with stages II, III, and IV in the 8th edition had worse 10-year OS. A similar pattern was seen for 10-year CSS in patients with stage III and IV in the 8th edition. However, patients with stage I and II in the 8th edition had better 10-year CSS (Table 3).

**Table 3:** Comparison of patient 10-years OS and CSS among clinical stages between the 7th and 8th edition.

**Stage**	**10-years OS**			**10-years CSS**		
	**7th edition**	**8th edition**	***p*-Value**	**7th edition**	**8th edition**	***p*-Value**
I	91.3%	91.4%	0.854	99.6%	99.6%	0.765
II	82.3%	80.8%	0.559	96.3%	97.1%	0.874
III	90.5%	73.7%	<0.05	99.1%	93.5%	<0.05
IV	78.1%	44.2%	<0.01	93.6%	64.4%	<0.01

**Figure 3:**
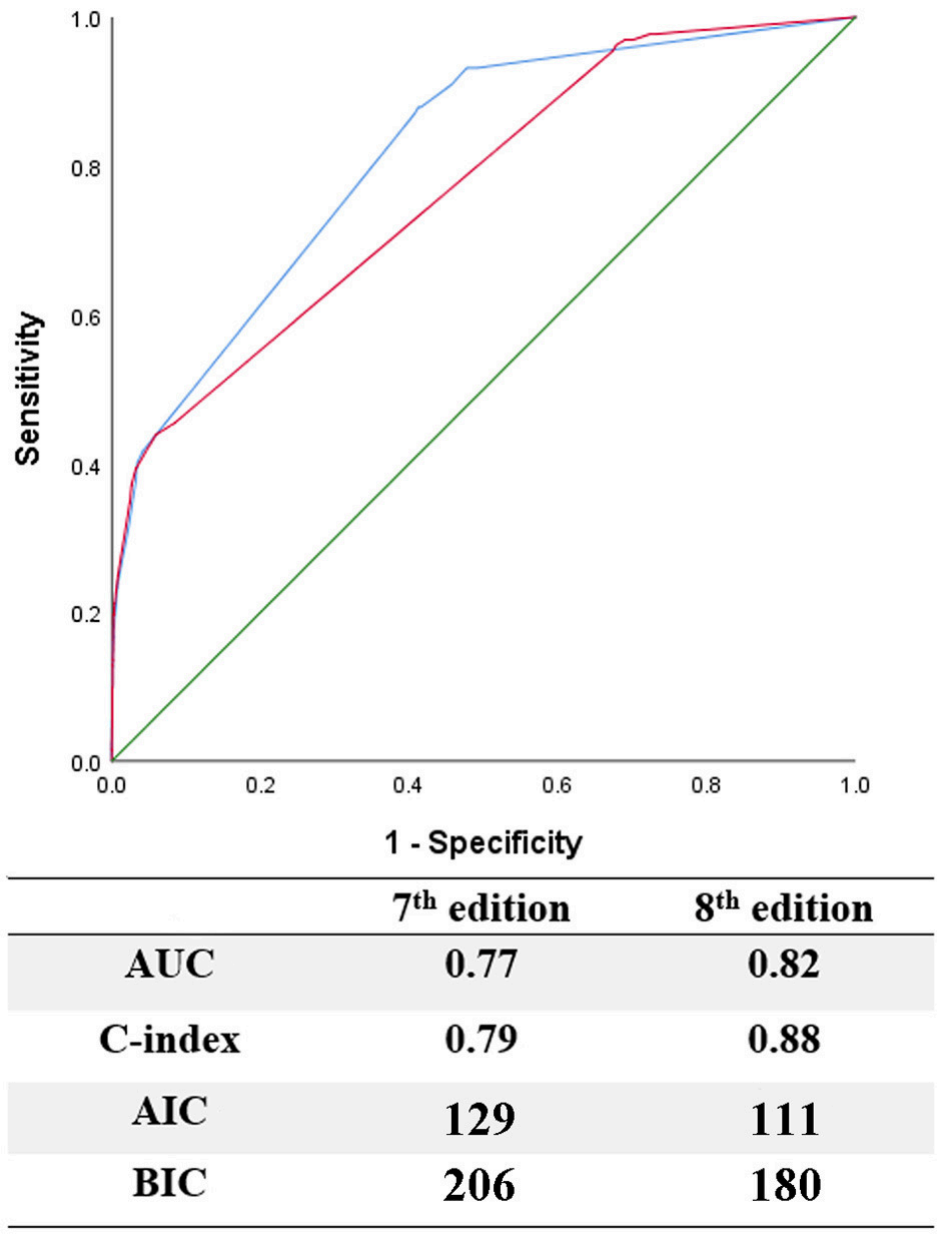
Evaluation of predictive performance between the 7th and 8th edition by using ROC curves, C-index, AIC, and BIC.

When evaluating the prognostic efficacy of the two editions, the 8th edition showed higher AUC and C-index, as well as lower AIC and BIC, thus indicating a better model performance than the 7th edition (Figure 3).

### Integrating ATA Risk Stratification With 7th and 8th Edition

According to the ATA risk stratification system, 33,428 (85.6%, all of whom were stage I in both editions) were classified as low-risk, 4,301 (11.0%) as intermediate-risk, and 1,303 (3.3%) as high-risk. When the ATA system was integrated with the AJCC/UICC staging system, the risk stratification changed, as follows: the intermediate-risk group shifted in stage I from 1,916 (44.5%) to 3,303 (76.8%), in stage II from 0 (0%) to 998 (23.2%), in stage III and stage IV both from 1649 (38.3%) and 736 (17.1%) to 0 (0%), respectively; the high-risk group shifted in stage I from 380 (29.2%) to 696 (53.4%), in stage II from 31 (2.4%) to 442 (33.9%), in stage III from 579 (44.4%) to 77 (5.9%) and in stage IV from 313 (24.0%) to 88 (6.8%).

**Figure 4:**
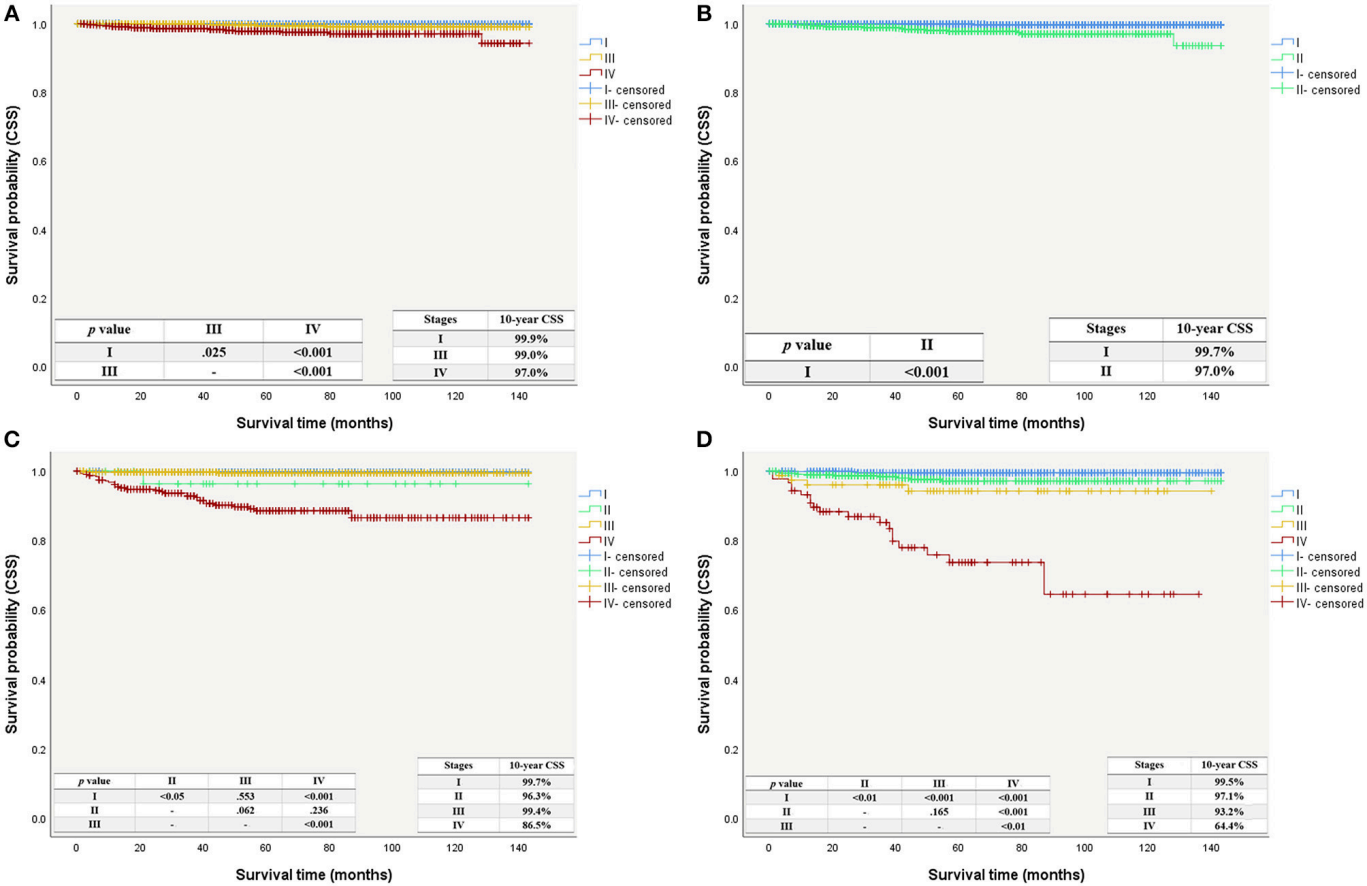
Cancer specific survival curves with patient using the AJCC/UICC staging system stratified by the ATA risk stratification system. **(A)** Cancer specific survival curves using the 7th edition stratified as intermediate-risk group. **(B)** Cancer specific survival curves using the 8th edition stratified as intermediate-risk group. **(C)** Cancer specific survival curves using the 7th edition stratified as high-risk group. **(D)** Cancer specific survival curves using the 8th edition stratified as high-risk group.

Even with the updated AJCC/UICC staging system, the composition of the low-risk group remained unchanged, and patients in this group had a 10-years CSS of 99.6%. The 10-years CSS decreased as patients' stage increased in the intermediate-risk group, in line with the changing trend of the high-risk group. The Kaplan-Meier survival curves still showed a better separation of the stage curves in the 8th edition, when compared to the 7th edition (Figure 4).

## Discussion

The purpose of this study was to compare the survival values and the prognostic efficacy between the 7th and 8th editions of the AJCC/UICC staging system for papillary thyroid microcarcinoma using the SEER database.

Although many patients with papillary thyroid microcarcinoma have favorable prognosis, part of them have worse prognosis with tumor recurrence or distant metastasis. Kazaure et al. reported that some PTMC variants may contribute to worse prognosis (15). Typical PTMC, as well as FVPTMC were reported to have excellent prognosis (16–18). In this study, we assessed data of patients with PTMC and FVPTMC from the SEER database. Since the HRs in Cox regression analyses, 10-years OS and CSS were all found without significant differences between PTMC and FVPTMC, we combined the data of PTMC and FVPTMC together rather than severally in our subsequent analyses.

In the 7th edition, tumors with extrathyroidal extension (ETE) were classified as T3, regardless of the range of the extension into the perithyroidal tissue. Advanced T categories have been consistently reported as risk factors for OS and CSS (19–21), while the lack of association between T3 categories with minimal ETE with worse survival was controversial (21–23). Consequently, microscopic/minimal perithyroidal tissue extension is no longer considered as a criterion for defining T3 in the 8th edition. Considering the new edition, about half of patients with advanced T categories were reclassified as early T stages in the current study. Dispense with considering tumor size, all patients who were reclassified as T3b, according to the 8th edition, in this study had extrathyroidal extension to the strap muscle, and all patients with minimal ETE were reclassified as T1a rather than T3. On the other hand, no significant differences in HRs were found between T1a and T3b patients. Patients with tumor extension code 450 (i.e., minimal extra thyroid extension, including strap muscles) were classified as T1a, and those with tumor extension code 480 (i.e., extension to the pericapsular soft tissue/connective tissue) were classified as T3b in this study. Due to the uncertain definitions of tumor extension codes in the SEER database, these reclassifications might cause downstaging of patients with gross strap muscle extension into T1a or upstaging of patients with minimal pericapsular extension into T3b. As strap muscles can be resected *en bloc* with the thyroid gland during surgery, it is still lack of further investigation as to why especially strap muscle extension is considered to be a greater risk otherwise than microscopic perithyroidal tissue extension.

In our study, according to the 8th edition, the N1a category did not lead to worse OS when compared to the N0 category, while N1b did it. As for CSS, our results were in accordance with a previous study assessing 10,000 PTC patients using the SEER database, which found that both N1a and N1b led to an increased risk of cancer-related death (24). Regarding the N categories, the new edition of the AJCC/UICC staging system de-emphasized the risk of superior mediastinal lymph node (level VII) metastasis, when compared to the 7th edition, as there were no obvious anatomical boundaries between the superior mediastinal (level VII) and central cervical (level VI) lymph nodes. As reported by a number of studies, cervical lymph node metastases can impair CSS, especially in older patients; however, their impact seems to be weaker when compared to advanced T categories (T4a/b) or distant metastases (M1). Furthermore, although lateral cervical lymph node metastasis contributes to worse prognosis, it is also reported that other characteristics, such as the metastatic lymph node size, number, and extra-nodal extension, also influence prognosis and still remain to be deeply investigated (25, 26).

Age at diagnosis is an important factor for survival in virtually all thyroid cancer staging systems. This non-anatomic factor, as a dichotomous variable, has been combined with other anatomic factors for staging thyroid cancer. Older patients were distributed among very advanced stages and had poorer prognosis. The age cutoff of 45 years old was used as a categorical variable in the 7th edition of AJCC/UICC staging system; however, this cutoff has been challenged. Mazurat et al. suggested the cutoff of 55 years as a better indicator of cancer-specific death risk (27). Moreover, after assessing 9,484 patients in a multicenter study, Nixon et al. reported that the age cutoff of 55 years old improved both the outcome prediction according to different stages and prognostic information (28). Our results support these findings, as patients changed from advanced to earlier cancer stages after the age cutoff was changed, and no markedly decreased OS or CSS were observed. Of note, several studies have reported higher risk with age as a continuous variable, and suggested using nomograms or multiple age classifications, instead of a single age cutoff, to predict the patient's survival risk (29, 30).

In our study, no statistically significant differences in OS and CSS were seen between patients with stages I and II, irrespective of the AJCC/UICC edition. However, patients with stage III and IV in the 8th edition had worse OS and CSS than those with the same stages in the 7th edition. The discriminative power of both editions to reveal survival was compared and our data suggests that the 8th edition is a better model than the 7th edition, as also evidenced by recently published reports (31, 32).

When integrated with the ATA risk stratification system, major changes were observed in the intermediate-risk and high-risk groups. The 8th edition showed a more precise risk stratification, especially for the high-risk group, as the survival curves showed a better separation of stages. A recent study showed a higher risk of persistence or recurrence in patients with PTC in advanced stages in the 8th edition (33). A study focusing on younger (under 55 years old) patients also found a higher mortality in patients from the high-risk group with early stages (stages I and II) (34) after integrating the 8th AJCC/UICC staging system and the ATA risk stratification system. These findings add to the improvements of the 8th edition to discriminate patients better in higher risk group and may remind physicians that the decrease of stages does not reflect less disease aggressiveness. Not only staging but also risk stratification should be assessed when caring patients with PTMC. However, other risk factors for recurrence, such as ^131^I-avid metastatic foci in the neck on the first post-treatment whole-body radionuclide scan (intermediate risk), incomplete tumor resection (high risk) and detection of elevated postoperative serum thyroglobulin suggestive of distant metastases (high risk), were not analyzed in our study due to the lack of available data in the SEER database (10).

This study has some notable limitations. First, the study probably had a selection bias, as its study focused on patients with PTMC and FVPTMC, and most cases were early-stage tumors. As we did not use the updated SEER database (which include follow-up data from 2015), the follow-up period for part of the patients assessed here may not have been sufficient to observe recurrence or cancer-specific death. Continuing surveillance of these patients is still necessary. Potential coding errors may not be ruled out, although the SEER database is standardized and appropriately audited. Finally, the current study did not include treatment information, as well as disease recurrence and novel outcome predictors, such as molecular markers, that were not included in the SEER database. Despite of these limitations, this study assessed a large cohort with a relatively long follow-up period, which valorize its contributions to the evaluation and comparison of the prognostic efficacies of the 7th and 8th editions of the AJCC/UICC staging system.

## Conclusion

In this study, we compared the prognostic values of the 7th and 8th versions of the AJCC/UICC staging system for patients with PTMC and FVPTMC and integrated them with the ATA risk stratification system. The 8th edition model provided a meaningful risk stratification and had a higher accuracy than the 7th edition, thus appearing to be superior to the 7th edition for evaluating patient survival.

## Author Contributions

JF and ML contributed to the conception and design of the work. FY and QZ participated to data analysis and text editing. FY and ML participated to data collection. JF and ZH contributed to text revision.

### Conflict of Interest Statement

The authors declare that the research was conducted in the absence of any commercial or financial relationships that could be construed as a potential conflict of interest.
