# Supplementary material for: Survival in Papillary Thyroid Microcarcinoma: A Comparative Analysis Between the 7th and 8th Versions of the AJCC/UICC Staging System Based on the SEER Database
Source: Front Endocrinol (Lausanne). 2019 Jan 24;10:10. doi: 10.3389/fendo.2019.00010 (PMC6354565; doi:10.3389/fendo.2019.00010)
Supplement: Supplementary file 1 [file Table_1.DOCX]

Supplementary Table1 (a)Brief summary of changes in age cutoff, primary tumor, lymph node metastasis. (b) TNM distribution into the 7^th^ and 8^th^ AJCC AJCC/UICC staging system.

(a)

| Changes in definition | Details | |
| --- | --- | --- |
| Patient age cutoff | For clinical staging purposes, patient age cutoff increased from 45-year-old to 55-year-old | |
| Primary tumor  (T category) | Minimal extrathyroidal extension was removed from staging. | |
|  | New T3 category: | T3a: Intrathyroidal tumor > 4cm |
|  |  | T3b: Any size tumor with gross extrathyroidal extension |
| Lymph node metastasis  (N category) | N1a now includes lymph node metastases in level Ⅵ and level Ⅶ, N1b includes lateral and retropharyngeal lymph node metastases | |
|  | pNx and pN0 are used interchangeably denoting lack of pathological metastasis | |

(b)

| Age cutoff | Stage | TNM grouping | |
| --- | --- | --- | --- |
|  |  | 7^th^ | 8^th^ |
| <45 years old(7^th^)/  <55 years old(8^th^) | Ⅰ | Any T, Any N, M0 | Any T, Any N, M0 |
|  | Ⅱ | Any T, Any N, M1 | Any T, Any N, M1 |
| ≥ 45 years old(7^th^)/  ≥ 55 years old(8^th^) | Ⅰ | T1, N0, M0 | T1-2, N0/Nx, M0 |
|  | Ⅱ | T2, N0, M0 | T1-2, N1, M0/  T3a/T3b, Any N, M0 |
|  | Ⅲ | T3, N0, M0/  T1-3, N1a, M0 | T4a, Any N, M0 |
|  | Ⅳa | T4a, N0-N1b, M0/  T1-3, N1b, M0 | T4b, Any N, M0 |
|  | Ⅳb | T4b, Any N, M0 | Any T, Any N, M1 |
|  | Ⅳc | Any T, Any N, M1 | - |
