# Supplementary material for: Survival in Papillary Thyroid Microcarcinoma: A Comparative Analysis Between the 7th and 8th Versions of the AJCC/UICC Staging System Based on the SEER Database
Source: Front Endocrinol (Lausanne). 2019 Jan 24;10:10. doi: 10.3389/fendo.2019.00010 (PMC6354565; doi:10.3389/fendo.2019.00010)
Supplement: Supplementary file 2 [file Table_2.DOCX]

Supplementary Table2 The TNM stage of patients in the 8^th^ AJCC/UICC staging system defined by SEER codes

| Stage | Code |
| --- | --- |
| T1a | Tumor extension codes 100, 200, 300, 400, 450 |
| T3b | Tumor extension codes 480, 490 |
| T4a | Tumor extension codes 500, 520, 550, 560, 600 |
| T4b | Tumor extension codes 620,700, 800 |
| N0 | Lymph node metastasis code 000 |
| N1a | Lymph node metastasis codes 120, 125, 158 |
| N1b | Lymph node metastasis codes 135, 155, 160, 170 |
| N1NOS (not otherwise specified) | Lymph node metastasis codes 180, 800 |
| M0 | Distant metastasis code 00 |
| M1 | Distant metastasis codes 12, 40, 51, 50 |
